# Is the combination of linagliptin and allopurinol better prophylaxis against post-contrast acute kidney injury? A multicenter prospective randomized controlled study

**DOI:** 10.1080/0886022X.2023.2194434

**Published:** 2023-03-28

**Authors:** Ahmed Fayed, Ahmed A. Hammad, Dina O. Abdulazim, Hany Hammad, Mohamed Amin, Samir Elhadidy, Mona M. Salem, Ibrahim M. Abd ElAzim, Lajos Zsom, Eva Csongradi, Karim M. Soliman, Usama A. Sharaf El Din

**Affiliations:** a Nephrology Unit, Internal Medicine Department, Kasr Alainy School of Medicine, Cairo University, Giza, Egypt; b Endocrinology Unit, Internal Medicine Department, Faculty of Medicine, Fayoum University, Faiyum, Egypt; c Rheumatology and Rehabilitation Department, Kasr Alainy School of Medicine, Cairo University, Giza, Egypt; d Critical Care Medicine Department, Internal Medicine Department, Kasr Alainy School of Medicine, Cairo University, Giza, Egypt; e Endocrinology Unit, Internal Medicine Department, Kasr Alainy School of Medicine, Cairo University, Giza, Egypt; f Critical Care Medicine, Theodor Bilharz Research Institute, Cairo, Egypt; g Fresenius Medical Care Hungary, Cegléd, Hungary; h Department of Medicine, Faculty of Medicine, University of Debrecen, Debrecen, Hungary; i Department of Surgery, Division of Transplant, Medical University of South Carolina, Charleston, SC, USA

**Keywords:** Post-contrast AKI, DPP4Is, linagliptin, allopurinol, N-acetyl cysteine

## Abstract

**Background:**

Patients with diabetic kidney disease (DKD) are at increased risk to develop post-contrast acute kidney injury (AKI). Diabetic patients under dipeptidyl peptidase 4 inhibitors (DPP4Is) experience a lower propensity to develop AKI. We speculated that linagliptin as a single agent or in combination with allopurinol may reduce the incidence of post-contrast AKI in stage 3–5 chronic kidney disease (CKD) patients with underlying DKD.

**Methods:**

Out of 951 DKD patients eligible for this study, 800 accepted to sign informed consent. They were randomly allocated to 4 equal groups that received their prophylaxis for 2 days before and after radiocontrast. The first control group received N-acetyl cysteine and saline, the 2^nd^ received allopurinol, the 3^rd^ group received linagliptin, and the 4^th^ received both allopurinol and linagliptin. Post-procedure follow-up for kidney functions was conducted for 2 weeks in all patients.

**Results:**

20, 19, 14, and 8 patients developed post-contrast AKI in groups 1 through 4, respectively. Neither linagliptin nor allopurinol was superior to N-acetyl cysteine and saline alone. However, the combination of the two agents provided statistically significant renal protection: post-contrast AKI in group 4 was significantly lower than in groups 1 and 2 (*p* < 0.02 and <0.03, respectively). None of the post-contrast AKI cases required dialysis.

**Conclusion:**

Linagliptin and allopurinol in combination may offer protection against post-contrast AKI in DKD exposed to radiocontrast. Further studies are needed to support this view.

**Trial registration ClinicalTrials.gov:**

NCT03470454

## Introduction

AKI is a common complication of radiocontrast exposure, especially in patients carrying underlying risk factors [1]. Among the non-modifiable risk factors, diabetes mellitus and CKD carry the highest risk [2]. The role of enhanced hypoxia and subsequent excess formation of reactive oxygen species (ROS) in the renal tissue following the administration of iodinated contrast media was demonstrated in many *in vitro* and *in vivo* studies [3]. A previous meta-analysis indicated that allopurinol might be an effective intervention compared with hydration and N-acetyl cysteine to prevent post-contrast AKI [4]. The preventive effect of allopurinol may be more remarkable in high-risk patients [5]. DPP4Is were not tried as preventive agents against post-contrast AKI. DPP4Is were found associated with a decreased risk of AKI among diabetic patients [6]. DPP4Is down-regulate the expression of the proinflammatory cytokines such as TNF*α*, IL-1*β*, IL-6, and chemokines such as MCP-1, hence; could be a potential mode of prevention of contrast-induced nephropathy [7].

In this randomized prospective study, we looked for the possible effect of linagliptin as a single agent or in combination with allopurinol to prevent post-contrast AKI in diabetic nephropathy patients.

## Methods

This trial was conducted between April 2018 and May 2020 in the Critical Care and Internal Medicine Departments of Cairo University, Fayoum University, and Theodor Bilharz Research Institute. The study protocol was revised and approved by the Ethics Committee of the Internal Medicine and Critical Care Departments at Cairo University, while the board review was approved by the Faculty of Medicine, Fayoum University research committee. Written consent was obtained from each patient or the patient’s next of kin. All procedures carried out in this study involving human subjects adopted the ethical principles of the Institutional Research Committee as well as the Helsinki Declaration of 1964 and its corresponding modifications or equivalent ethical standards. The trial registration number at ClinicalTrials.gov was NCT03470454.

We excluded patients who met any of the following criteria: those on other DPP4 inhibitors, glucagon-like peptide receptor agonists, sodium-glucose transporter-2 inhibitors; already on long-time linagliptin, febuxostat, or allopurinol therapy; those with a low HbA1c (<7%) due to concerns about possible hypoglycemic events and patients with heart failure.

The eligible patients were at least 30 years of age. All patients were maintained on statin treatment as part of their standard of care treatment. Metformin, renin-angiotensin-aldosterone antagonists, and diuretics were stopped once the patients were recruited to the study and reinstituted after the final analysis. 800 patients were randomized according to the type of radiologic intervention into four groups using Adaptive Randomization (outcome-adaptive randomization program for clinical trials from the M.D. Anderson Cancer Center, University of Texas).

**Figure 1.**
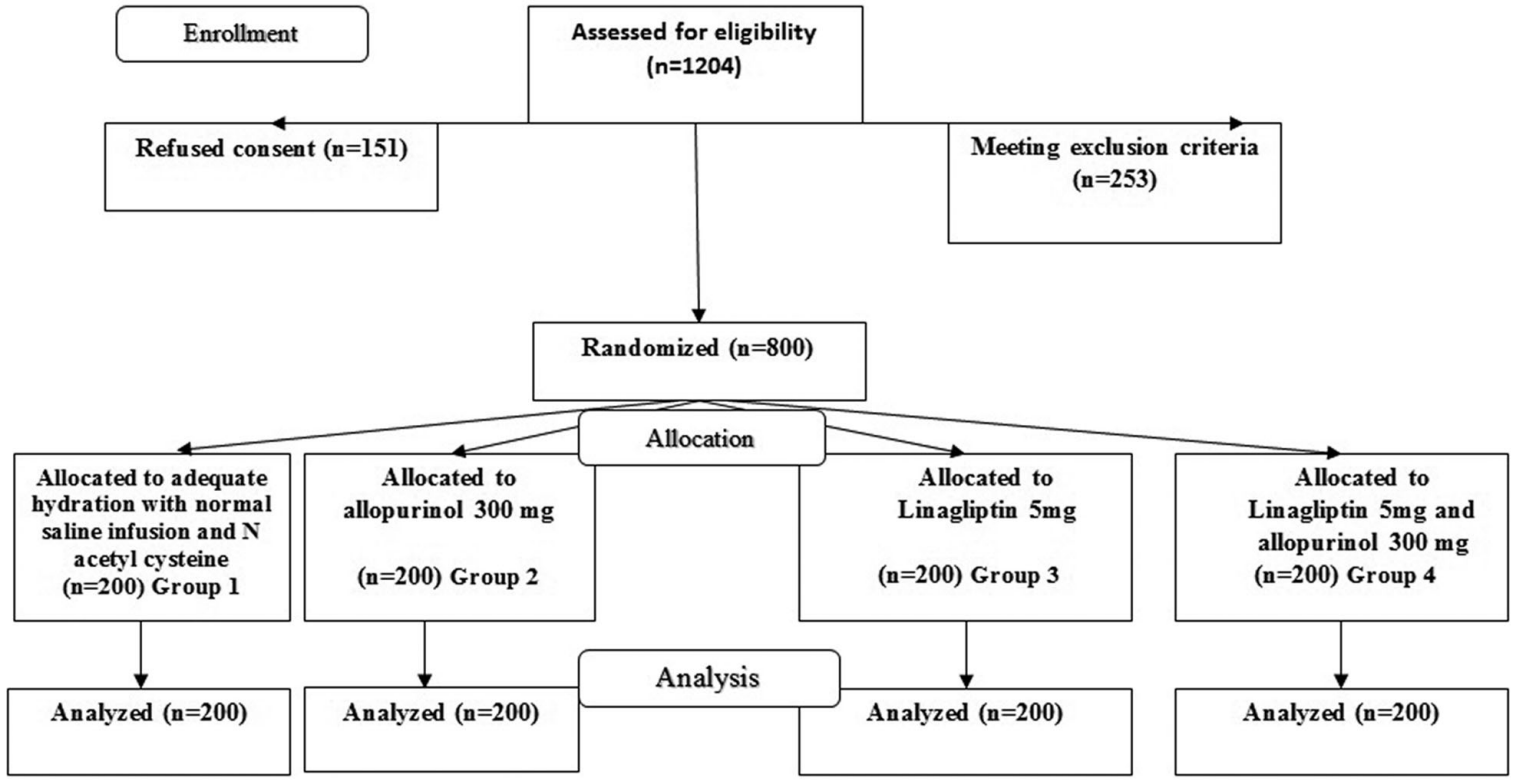
Study flow chart.

All the patients received the planned intervention 48 h before and 48 h after the radiocontrast administration. Baseline serum creatinine was obtained 72 h before the planned intervention and before the administration of any protective protocol. Follow-up serum creatinine was obtained 72 h after contrast administration. Group 1 was given 200 mg of N-acetyl cysteine orally every eight hours, as well as 100 mL/h of 0.9 g/dL saline solution 6 to 12 h before and after the contrast imaging technique, or 1 to 1.5 mL/kg/h of saline solution for 12 h before and up to 24 h after the procedure. Subjects in group 2 received 300 mg of allopurinol daily [5], group 3 received linagliptin 5 mg daily [7] and group 4 received linagliptin 5 mg and allopurinol 300 mg daily (Figure 1).

The baseline demographic, clinical characteristics, and initial laboratory investigations that were collected are presented in Table 1.

**Table 1.** Demographic and baseline laboratory data of the studied patients.

Variables		Group 1 (*n* = 200)	Group 2 (*n* = 200)	Group 3 (*n* = 200)	Group 4 (*n* = 200)	*p* Value
Age (Years) (Mean ± SD)		48.9 ± 7.3	49 ± 7.6	48.04 ± 6.5	48.98 ± 6.9	0.47
BMI (Kg/m^2^) (Mean ± SD)		25.3 ± 1.9	24.6 ± 2.7	26.4 ± 1.9	25.5 ± 1.7	<0.00001^a^
Smokers (Number (%))		101 (50.5)	99 (49.5)	121 (60.5)	97 (48.5)	<0.00001^a^
Hypertension (Number (%))		118 (59)	125 (62.5)	137 (68.5)	126 (63)	0.014^a^
Duration of Diabetes mellitus (Years) (Mean ± SD)		7.09 ± 3.5	7.15 ± 2.2	6.4 ± 1.5	6.9 ± 1.9	0.0188^a^
Laboratory data before contrast						
S. Urea (mg/dL) (Mean ± SD)		66.1 ± 21.8	76.9 ± 21.04	70.3 ± 22.4	74.6 ± 22.7	0.002^a^
Creatinine (mg/dL) (Mean ± SD)		2.4 ± 0.4	2.4 ± 0.4	2.4 ± 0.4	2.6 ± 0.5	0.001^a^
Estimated GFR (mL/min/1.73 m²) (Mean ± SD)		28.3 ± 6.7	28.5 ± 7.9	27.7 ± 7.4	24.8 ± 6.6	0.001^a^
Stage of CKD	Stage 3 CKD (Number (%))	69 (34.5)	71 (35.5)	68 (34)	40 (20)	0.0005^a^
	Stage 4 CKD (Number (%))	131 (65.5)	127 (63.5)	132 (66)	156 (78)	
	Stage 5 CKD (Number (%))	0 (0)	2 (1)	0 (0)	4 (2)	
Urine ACR (mg/g) (Mean ± SD)		113.6 ± 29.4	116.2 ± 30.1	113.3 ± 32.9	116.5 ± 33.7	0.714
Uric acid (mg/dl) (Mean ± SD)		6.4 ± 1.2	7.7 ± 0.6	5.5 ± 1.4	7.7 ± 0.7	0.0001^a^
HbA1c (Mean ± SD)		6.5 ± 0.4	6.5 ± 0.4	6.4 ± 0.4	6.4 ± 0.4	0.064
Patients with HbA1c ≤6.5% (Number (%))		129 (64.5)	101 (50.5)	118 (59)	113 (56.5)	

*P*-value calculated by one-way ANOVA calculator.^a^The result is significant at *p*< .05. BMI: body mass index; SD: Standard Deviation; GFR: glomerular filtration rate using MDRD equation; ACR: albumin/creatinine ratio; HbA1c: glycated hemoglobin.

The primary endpoint was the development of post-contrast AKI, defined as a decrease of GFR by or greater than 30% relative to baseline or an increase in serum creatinine that is greater than 0.3 mg/dl relative to baseline or 30% over baseline 72 h after the administration of the contrast. A secondary endpoint was the maximum absolute change in serum creatinine and GFR during the study period. GFR was estimated using the MDRD 4-variable GFR Equation (GFR in mL/min per 1.73 m^2^ = 175 x SerumCr^−1.154^ x age^−0.203^ x 1.212 (if the patient is black) x 0.742 (if female)). Table 2 summarizes the different radiologic procedures performed as well as the types and amounts of radiocontrast agents used. Change in any of the studied parameters was calculated as a change in percentage [{(post-level-basal level)/basal level} X 100].

**Table 2.** Details of the radio-contrast provided to the patients in the study, as well as laboratory data 72 h after the contrast.

Variables	Group 1 (*n* = 200)	Group 2 (*n* = 200)	Group 3 (*n* = 200)	Group 4 (*n* = 200)	*p* Value
Type of imaging with contrast					
Therapeutic Coronary Angiography (Number (%))	74 (37)	72 (36)	71 (35.5)	72 (36)	0.82887
Diagnostic Coronary Angiography (Number (%))	43 (21.5)	37 (18.5)	59 (29.5)	56 (28)	
CT Coronary Angiography (Number (%))	21 (10.5)	25 (12.5)	26 (13)	21 (10.5)	
High Resolution CT Chest (Number (%))	44 (22)	40 (20)	34 (17)	36 (18)	
CT Abdomen (Number (%))	18 (9)	26 (15)	10 (5)	15 (7.5)	
Type of the nonionic contrast used					
Iohexol (Omnipaque) (Number (%))	105 (52.5)	91 (45.5)	95 (47.5)	97 (48.5)	0.99987
Iopromide (Ultravist) (Number (%))	95 (47.5)	109 (54.5)	105 (52.5)	103 (51.5)	
Volume of contrast					
Volume (mL) (Mean ± SD)	114.5 ± 30.2	115.1 ± 29	112.6 ± 31.2	111.4 ± 30.9	0.58773
75mL (Number (%))	42 (21)	33 (16.5)	53 (26.5)	55 (27.5)	
100mL (Number (%))	79 (39.5)	90 (45)	70 (35)	72 (36)	
150mL (Number (%))	79 (39.5)	77 (38.5)	77 (38.5)	73 (36.5)	
Laboratory data 72 h after contrast injection using the Kruskal-Wallis test					
Post-contrast AKI (Number (%))	20 (10)	19 (9.5)	14 (7)	8 (4)	0.092
Percent change in urea (Mean ± SD)	−11.5 ± 10.6	−6.1 ± 4.6	−16.9 ± 10.2	−21.4 ± 11.3	<0.0001^a^
Percent change in creatinine (Mean ± SD)	−1.9 ± 10.3	−0.15 ± 9.7	−2.01 ± 10.7	−6.3 ± 6.8	0.0033^a^
Percent change in GFR (Mean ± SD)	3.6 ± 12	1.3 ± 11.3	3.8 ± 11.8	7.5 ± 11.01	<0.0001^a^
Percent change in uric acid (Mean ± SD)	−8.03 ± 8.8	−18.4 ± 4.4	−5.3 ± 7.2	−22.5 ± 6.2	<0.0001^a^
Percent change in Urine ACR (Mean ± SD)	−4.4 ± 3.4	−6.7 ± 5.6	−19.2 ± 16.2	−24.4 ± 14.4	<0.0001^a^
Post contrast AKI (Chi square,confidence interval 95%)					

**Table:** 

	Post contrast AKI (Number (%))	95% CI	*p* Value
Group 4 Contrast Induced Nephropathy (Number 8 (4%))			
Group 1(Number (%))	20 (10)	0.9577% to 11.3150%	0.0188^a^
Group 2(Number (%))	19 (9.5)	0.5247% to 10.7403%	0.0286^a^
Group 3(Number (%))	14 (7)	−1.6255% to 7.8216%	0.1888
Group 3 Contrast Induced Nephropathy (Number 14 (7%))			
Group 1(Number (%))	20 (10)	−2.5851% to 8.6714%	0.2827
Group 2(Number (%))	19 (9.5)	−3.0246% to 8.1014%	0.3641
Group 1 (*n* = 200) Contrast Induced Nephropathy (Number20 (10%))			
Group 2(Number (%))	19 (9.5)	−5.4503% to 6.4601%	0.8663

*P*-value calculated by one-way ANOVA calculator. ^a^The result is significant at *p*< .05. SD: Standard Deviation; CT: computerized tomography; GFR: glomerular filtration rate using MDRD equation; ACR: albumin/creatinine ratio.

### Statistical analysis

The data collected were verified, coded, entered, and analyzed with IBM Statistical Package for Social Science (SPSS) Statistics 22 software. The mean and standard deviation of continuous variables were calculated. For qualitative variables, frequency and percentage were used. The Mann-Whitney test was used to compare groups. A comparison between more than 2 independent groups was evaluated using the Kruskal-Wallis test (Table 2). For qualitative data, bivariate associations were examined using the chi-square test. P-values < 0.05 were considered statistically significant.

## Results

Patients selected for this study carry a very high risk to develop AKI upon exposure to radiocontrast. According to the risk score proposed by Mehran et al. [8] and updated by Barrett and Parfrey, [9] the total risk score to develop post-contrast AKI in the studied patients ranged between 6 and 12.

Demographic and baseline laboratory data of the studied patients were summarized in Table 1. The significant discrepancy in body mass index and kidney function at entry was not intentional. Consequently, we relied on the differences in the individual laboratory parameters before versus after radiocontrast administration (Table 2). Twenty cases (10%) in group 1, nineteen (9.5%) in group 2, fourteen (7%) in group 3, and eight (4%) in group 4 matched the definition of post-contrast AKI. There is no significant difference in the incidence of post-contrast AKI between group 3 versus group 1, group 2, or group 4 (Table 2).

Following the administration of renoprotective medications, the distinct groups showed a slight improvement in kidney function tests. The improvement in serum urea was the most evident in all groups and was most pronounced in group 4. Here, the percentage of decline in blood urea nitrogen was significantly greater than in the other three groups. The improvement in serum creatinine was the least in group 2, while the improvement in the glomerular filtration rate was maximal in group 3. On the other hand, a maximal antiproteinuric effect was observed in group 4 (Table 2). The significant hypouricemic effect is likely related to the use of allopurinol in groups 2 and 4.

## Discussion

In addition to diabetes, preexisting CKD is the strongest risk factor for the development of post-contrast AKI [10–12]. Although periprocedural intravenous crystalloid infusion is still the primary intervention recommended by the American College of Radiology and the European Society of Cardiology to mitigate the risk of post-contrast AKI, this approach carries considerable risk to patients with underlying heart disease or systemic hypertension [13]. N-acetyl cysteine is usually used together with isotonic saline in post-contrast AKI prevention protocols due to its known antioxidant effect, low cost, ease to use, and appreciable safety. When N-acetyl cysteine was used without hydration therapy, it did not reduce the risk of post-contrast AKI [14], while its addition to saline led to conflicting results [15,16]. When allopurinol was used instead of acetylcysteine in several trials, it potentiated the renoprotective effect of hydration therapy [5,17,18]. This effect is probably consequent to the mitigation of the stimulatory effect of intracellular uric acid on nicotinamide adenine dinucleotide phosphate (NADPH) oxidase. The activation of NADPH oxidase causes increased intracellular oxidative stress, mitochondria injury, ATP depletion, and the activation of nuclear factor kappa-B (NF-κB) [19,20]. We did not encounter studies of allopurinol without hydration therapy. Although the use of DPP4Is is associated with a decreased incidence of AKI among diabetic patients [6], the literature lacks studies on the possible renoprotection these agents can offer to patients exposed to radiocontrast. The present study aimed to look for the preventive effect of DPP4I linagliptin in comparison to standard periprocedural hydration plus N-acetyl cysteine, allopurinol, or the combined use of linagliptin and allopurinol. DKD with overt proteinuria is often accompanied by avid sodium retention [21]. Hence, it seems unlikely that DKD patients need fluid infusion to prevent post-contrast AKI. Based on this assumption, we did not add fluid therapy to linagliptin or allopurinol. The results of the current study confirm that neither linagliptin nor allopurinol is inferior to intravenous fluid therapy. Moreover, the combination of linagliptin and allopurinol was superior to both fluid therapy and the separate use of these two agents. Accordingly, this is the first study to demonstrate the applied prophylactic therapy implying synergism between these two agents. In animal studies, DPP4Is significantly reduce the markers of tubular necrosis and proinflammation markers in uninephrectomized rats exposed to ischemia-reperfusion injury [22,23]. These anti-inflammatory and anti-apoptotic actions of linagliptin, when combined with the antioxidant impact of allopurinol, could result in a renoprotective effect. Lastly, the results of the present study should interrupt the long-term inertia in the field of post-contrast AKI prevention [24]. The small sample size & short period of observation in the current investigation were the major limitations of the study. This study is an uncontrolled observational cross-sectional study, and as such, its findings may contain biases that are challenging to identify or correct. Future studies should verify the results of this study, look into the effects of higher doses of either allopurinol or DPP4I versus the combination of these two agents, and explore novel antioxidant, anti-inflammatory, and anti-apoptotic medications, either independently or in combination.

## Conclusion

Linagliptin and allopurinol in combination may offer protection against post-contrast AKI in DKD exposed to radiocontrast. Further studies are needed to support this view.
